# IPANEMAP Suite: a pipeline for probing-informed RNA structure modeling

**DOI:** 10.1093/nargab/lqaf028

**Published:** 2025-03-25

**Authors:** Pierre Hardouin, Nan Pan, Francois-Xavier Lyonnet du Moutier, Nathalie Chamond, Yann Ponty, Sebastian Will, Bruno Sargueil

**Affiliations:** CNRS UMR 8038, CiTCoM Cibles Thérapeutiques et Conception de Médicaments, Université Paris Cité, 4 avenue de l’Observatoire, 75270 Paris, France; CNRS UMR 7161, LIX, Ecole Polytechnique, 1 rue Estienne d’Orves, 91120 Palaiseau, France; CNRS UMR 8038, CiTCoM Cibles Thérapeutiques et Conception de Médicaments, Université Paris Cité, 4 avenue de l’Observatoire, 75270 Paris, France; CNRS UMR 8038, CiTCoM Cibles Thérapeutiques et Conception de Médicaments, Université Paris Cité, 4 avenue de l’Observatoire, 75270 Paris, France; CNRS UMR 7161, LIX, Ecole Polytechnique, 1 rue Estienne d’Orves, 91120 Palaiseau, France; CNRS UMR 7161, LIX, Ecole Polytechnique, 1 rue Estienne d’Orves, 91120 Palaiseau, France; CNRS UMR 8038, CiTCoM Cibles Thérapeutiques et Conception de Médicaments, Université Paris Cité, 4 avenue de l’Observatoire, 75270 Paris, France

## Abstract

In addition to their sequence, multiple functions of RNAs are encoded within their structure, which is often difficult to solve using physico-chemical methods. Incorporating low-resolution experimental data such as chemical probing into computational prediction significantly enhances RNA structure modeling accuracy. While medium- and high-throughput RNA structure probing techniques are widely accessible, the subsequent analysis process can be cumbersome, involving multiple software and manual data manipulation. In addition, the relevant interpretation of the data requires proper parameterization of the software and a strict consistency in the analysis pipeline. To streamline such workflows, we introduce IPANEMAP Suite, a comprehensive platform that guides users from chemically probing raw data to visually informative secondary structure models. IPANEMAP Suite seamlessly integrates various experimental datasets and facilitates comparative analysis of RNA structures under different conditions (footprinting), aiding in the study of protein or small molecule interactions with RNA. Here, we show that the unique ability of IPANEMAP Suite to perform integrative modeling using several chemical probing datasets with phylogenetic data can be instrumental in obtaining accurate secondary structure models. The platform’s project-based approach ensures full traceability and generates publication-quality outputs, simplifying the entire RNA structure analysis process. IPANEMAP Suite is freely available at https://github.com/Sargueil-CiTCoM/ipasuite under a GPL-3.0 license.

## Introduction

The integration of chemical structure probing experiments significantly improves the accuracy of computations for predicting RNA secondary structure. The information from such experiments, by and large, position-wise pairing propensities, is integrated into folding algorithms as soft constraints [1–3]. Typically, these propensities are determined as reactivities with specific chemical probes. For example, Deigan *et al.* [1] transform the reactivities into pseudo-energies, which specifically penalize or reward the pairing of the individual nucleotides in predicted structures. Different types of probing experiments were proposed, varying in their chemistry and readout of the structure-informing reaction rates of these probes with RNA nucleotides. Concerning the former, one distinguishes two main types of chemical probes: first, DMS (dimethyl sulfate) or CMCT [cyclohexyl-3-(2-morpholinoethyl) carbodiimide metho-*p*-toluene sulfonate], which probe the availability of H-bond donor or acceptor sites located on the Watson–Crick face of the base [4]; second, SHAPE probes that report on the flexibility of the ribose, reacting with its 2′ hydroxyl group [5–8].

Eventually, both types of probes induce covalent modifications of the nucleoside with structure-dependent frequencies. These modifications can be read out and converted to reactivity scores. The modifications are mapped using reverse transcriptase (RT), where they lead to the premature termination of the reverse transcription.

Such experiments are analyzed on capillary electrophoresis (CE) systems.

Several pipelines have been developed to analyze the electrophoresis results yielding a reactivity profile for the RNA of interest [9–13]. Reactivity profiles can then be fed to RNA secondary structure prediction software, such as RNAFold [14, 15], RNAstructure [16, 17], or Shapeknot [18], to guide the prediction.

However, the existing pipelines were developed to utilize the information from single probing experiments (including replicates), while recent results promise strong benefits from combining results of multiple probing experiments in the modeling of one RNA [19–23]. For example, experiments can vary in the use of chemical probes and specific experimental conditions (salts, temperature, etc). Since they can provide complementary information, integrating multiple reactivity profiles at the same time has the potential to substantially improve the modeling accuracy.

For instance, our recently developed bioinformatics tool IPANEMAP [23] was shown to match the performance and even outcompete available structure modeling tools due to clean integration of multiple probing data.

The development of such an innovative modeling workflow eased and enhanced the accuracy of experimentally driven RNA secondary structure modeling. However, using them requires the local installation of several in-house developed software or scripts with different prerequisites and to run each script manually through the use of command lines. In addition, going from the raw CE files to the RNA secondary structure drawing colored with reactivities involves cumbersome file manipulations from one software to another, often requiring format adaptation and calculations. The whole process ends up being very time-consuming, complex, and error-prone, especially for inexperienced users. To prevent such problems, convenient web servers, such as RNAprobe [24] or RNAthor [25], have been developed, but none support an initial processing of CE data, or offer the possibility to integrate datasets obtained with different probes.

We developed IPANEMAP Suite to fill this gap while ensuring a full traceability and reproducibility all along the process. It is designed as a fully integrated and highly configurable pipeline organizing the user journey from CE files to the RNA secondary structure model representation.

IPANEMAP Suite is grounded on a new version of IPANEMAP that offers the possibility to exploit the covariation information contained in a multiple sequence alignment (MSA) of a phylogeny of the RNA of interest, further improving its prediction performance. CE file analysis is provided by QuShape [12], but structure probing data from other CE data processing software [9–13], or from high-throughput “Map” or “Seq” technologies, such as SHAPE-MaP [26], SHAPE-Seq [27, 28], and DMS-MaPseq [29], can be readily fed into the pipeline. Finally, it is known that many functions performed by RNA hinge critically on its capacity to interact specifically with proteins or small molecule metabolites and/or to adapt its structure to environmental variations, such as ionic conditions, temperature, or pH. RNA structural flexibility and/or intermolecular interactions can efficiently be monitored with probing technologies [20, 30, 31]. Indeed, the susceptibility of each nucleotide to chemical probes can be determined under different experimental conditions or in the absence/presence of a ligand. Interpreting such data requires a systematic experimental and analytical workflow as well as the application of objective and statistical criteria. IPANEMAP Suite implements a “footprinting” framework allowing to compare and interpret chemical reactivity maps obtained in different conditions or in the presence of an RNA ligand.

## Materials and methods

### Structure probing

RNA structure probing was carried out as previously described in [32, 33].

### Input file format

IPANEMAP Suite accepts input as raw ABI and FSA files from sequencers (Applied Biosystems), as well as output from the CEQ8000/genomeLab (SCIEX) to derive reactivity profiles. Moreover, it directly imports various reactivity files from QuShape or other analysis software, including those from HTS technologies, such as SHAPE-MaP, SHAPE-seq, and DMS-MaPseq. Files accepted are tab-delimited text, including the nucleotide number in the first column and its reactivity in the second. Sequences and MSA files are read in fasta format (.fas). Although input files can be named arbitrarily, following a defined naming convention facilitates the integration into the pipeline (see Supplementary data for a tutorial).

### Removal of outliers and normalization

Outliers corresponding to intrinsic RT stops are removed as previously described in [33, 34]. Two normalization methods are implemented in IPANEMAP Suite: The first “simple” method takes the 2% of the most reactive peaks of all reactivities (modified and unmodified conditions) and removes them; they empirically correspond to intrinsic stops. Then, the 8% of the most reactive peaks are averaged, and the reactivities are normalized by this average. The second “interquartile” method uses parametric removal: peaks whose value is higher than the 75th percentile by at least 1.5 times the interquartile range are considered as outliers and removed. The 10% of the remaining higher values are averaged and the average is used to normalize all reactivities. Based on empirical observations, the “simple” method (used by default) is recommended for molecules shorter than 300 nt, and the interquartile method is typically reserved for longer molecules [34]. The normalization method can be parameterized by the user, and IPANEMAP Suite enforces the use of only a single method per project in order to ensure consistency. Finally, negative values down to −0.3 are set to zero, while inferior negative values are considered as undetermined and set to −10 to be interpreted as missing by subsequent analyses.

### Data aggregation and consistency checks

One of the key features of the IPANEMAP Suite pipeline is its ability to use multiple datasets as constraints for predictive modeling. When multiple datasets are available for the same RNA under identical probing conditions, IPANEMAP Suite treats them as replicates and aggregates their reactivity data.

Our aggregation strategy is designed to work well with the typically low numbers of replicates. Therefore, it resembles steps of a typical manual aggregation to produce interpretable results.

In detail, IPANEMAP Suite proceeds position by position, as follows: First, positions with too few valid values are labeled as “undetermined”.

For every other position, IPANEMAP Suite computes the mean and standard deviation of its reactivities. By default, positions are accepted and the mean is assigned as an aggregation result, if one of two criteria is satisfied:

- the standard deviation of the reactivities of the replicates at this position is less than or equal to min_std (defaulting to 0.15);
- all pairwise means of reactivities fall in the same reactivity range as the total mean, distinguishing the following reactivity ranges: below reactivity_medium, between reactivity_medium, and reactivity_high, and above reactivity_high (with defaults reactivity_medium = 0.4 and reactivity_high = 0.7).

Otherwise, no reactivity is used at this position in the downstream modeling process. Such positions are either marked as ‘‘nonconsistent’’ or “warning”, and are shown in the color-coded structure representations. For this purpose, we flag reactivities as “warning”, if all pairwise means deviate from the overall mean but still fall within a consistent range.

While this specific data treatment works well for us with default values min_std = 0.15, reactivity_medium = 0.4, and reactivity_high = 0.7, these parameters can be modified in the project configuration. This allows the user to turn off the data treatment and use all aggregated reactivities for structure modeling.

Independently, at this stage, it is possible to assay the overall consistency between two replicates by calculating pairwise Pearson and Spearman correlation coefficients. This allows for a fast identification of failed experiments to exclude them from the project.

#### Concatenation of data obtained with different primers on the same RNA

IPANEAMAP Suite seamlessly supports the use of multiple primers per RNA as typically performed when probing long RNAs. In this case, the originally separate reactivity profiles of the same probing condition are properly aggregated and merged into single profiles of the RNA. This alleviates tedious file manipulation and a potential source of error.

### IPANEMAP: multiple probing-informed prediction

RNA secondary structures are predicted using IPANEMAP (v0.1.9) [23], based on the dataset(s) generated in previous steps. A single project can initiate arbitrarily many IPANEMAP predictions with different combinations of input data and configurations. For each prediction, the IPANEMAP Suite generates two types of RNA secondary structure outputs: a standard dot–bracket file allowing you to draw the secondary structure with most of the secondary structure visualization software and a VARNA session file allowing the visualization of the secondary structure colored according to the reactivity of each nucleotide with the probe. Note that if several probes or conditions are used by IPANEMAP, a VARNA file colored with each reactivity set is generated.

#### IPANEMAP core functionality

IPANEMAP was specifically designed to integrate several reactivity profiles from experiments under different conditions into the modeling of RNA secondary structures. It performs this task by sampling structures informed by each of the input reactivity data, followed by a clustering of the structures. It predicts structures based on their occurrence in large, favorable clusters supported by multiple conditions [23]. Roughly, IPANEMAP extends and generalizes the structure sampling and clustering-based prediction approach of Sfold [35] to probing-informed prediction under potentially multiple probing conditions.

#### Integration of phylogenetic data

For IPANEMAP Suite, the tool IPANEMAP (v0.1.9) was extended to make use of phylogenetic data, in order to additionally support structure modeling. The use of this option needs to be declared in the configuration step, and the MSA fasta file must be placed manually in the ‘‘resources’’ folder as for sequence files. This option was implemented in IPANEMAP using RNAalifold [14, 36], which predicts consensus structures of the given alignment. These predictions are then treated analogously to predictions based on a different reactivity profile. Effectively, the alignment is integrated in the same way as data from additional probing condition.

### Footprinting analysis

The probing profiles obtained in two different conditions are compared as described in [37, 38]. In brief, the reactivities for each nucleotide in two different conditions are compared using their difference, their relative difference (difference/sum), and a *t*-test.

In more detail, the reactivities averaged from three replicates in two conditions, *R*_1_ and *R*_2_, were used to calculate the absolute difference Δ*R* = |*R*_1_ − *R*_2_| and the relative difference ${\Delta R}/({R_{1} + R_{2}})$. The reactivity difference between two conditions is considered significant when both variables exceed a threshold of 0.2, and the *P*-value from a two-sided *t*-test is inferior to .05. Nucleotides with undetermined reactivity are excluded from the analysis. Such an approach excludes small reactivity changes between weakly reactive nucleotides as well as large yet unmeaning reactivity changes between highly reactive nucleotides.

### Visualization of candidate structures

IPANEMAP Suite uses VARNA (v0.94.1) to draw RNA secondary structures in the visualization of IPANEMAP predictions and footprinting results. The color annotation capabilities of VARNA were extended to annotate nucleotides according to their reactivity class (undefined, low, medium, and high). In footprinting analyses, this feature allows us to highlight differentially reactive positions in the context of predicted structures. Moreover, within structural comparison, it allows for the representation of bases that share the same or differ in structure between two models, as well as paired bases with different partners in the two structures.

## Results

### Detailed overview of the IPANEMAP Suite

IPANEMAP Suite is a one-stop solution to model RNA secondary structure from output files of CE of a probing experiment to the representation of the secondary structure colored according to the reactivity of each nucleotide. It may also integrate probing data from HT probing technologies, such as ShapeMap, ShapeSeq, or DMS-MaPseq.

**Figure 1.**
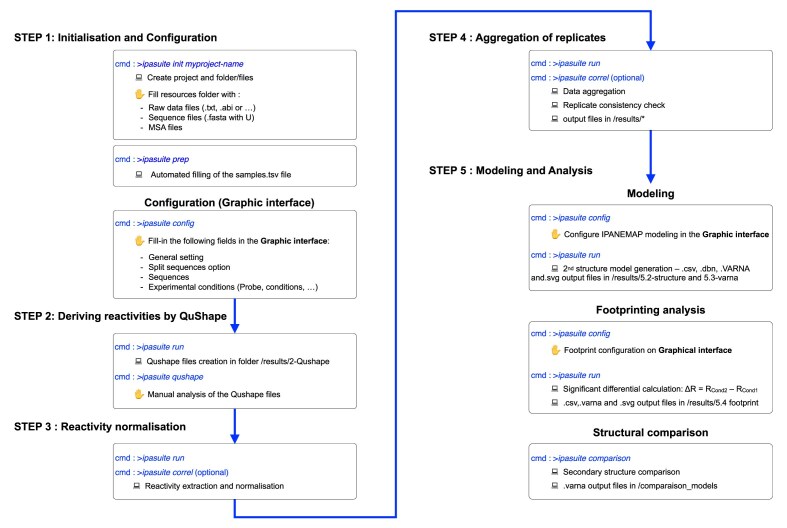
The project-centered workflow of IPANEMAP Suite: a visually intuitive interface for configuring the entire chemical probing data analysis pipeline within IPANEMAP Suite. Users can easily customize each step of the process, from data import and preprocessing to analysis and visualization.

IPANEMAP Suite moreover avoids dependency and potential compatibility problems, facilitates maintenance, and ensures reproducibility of the results. IPANEMAP Suite has been designed to be user-friendly and prevent tedious file manipulation from one software to another. It is project-oriented and allows for full traceability all along the project, preventing any mix-up or misinterpretation. A project may include different RNA sequences, probed in different experimental conditions performed by one or several experimentalists. It allows modeling RNA structure using different sets of data obtained with different probes or in different conditions following the process of the IPANEMAP workflow [19, 23]. IPANEMAP Suite additionally implements functionality to incorporate the phylogenetic information contained in a MSA in the modeling process. It outputs two RNA secondary structure models for each RNA sequence and for each series of conditions chosen by the user in the configuration interface. Granted that the two secondary structure models belong to two different structural clusters, they may represent alternative foldings. IPANEMAP Suite may also analyze and highlight the significant differences between two probing conditions if the footprint option is run. Finally, the function “comparison” colors secondary structure models for a fast visual comparison of two models of the same RNA. The software is packaged using Conda to simplify its installation on Linux, or Windows (using WSL), or MacOS. All requirements and steps to install and run IPANEMAP Suite are described in detail at https://sargueil-citcom.github.io/ipasuite-docs. In the following, we describe the main stages performed by IPANEMAP Suite step by step; see Fig. 1 for a brief overview.

#### Step 1: initialization of projects and configuration

##### Initialization of IPANEMAP Suite projects

Projects are created and initialized using a single command (ipasuite init myproject_name; see Fig. 1). Upon initialization, files and folders are necessary to run the project (see Supplementary Fig. S2). Raw data files, sequence fasta files, MSA, and/or reactivity files may then be added in the resources folder (see Supplementary Fig. S2).

##### User-friendly configuration

IPANEMAP Suite offers a user-friendly, web-based interface for configuring sequences, probing reagents, probing conditions, IPANEMAP settings, and footprinting analysis options. IPANEMAP Suite offers the possibility to automatically concatenate results obtained from the same sequence using different primers. This facilitates the work with long RNAs and eliminates a source of error. Any information can be updated throughout the progression of the project, allowing the analysis pipeline to operate with the initial results and the integration of further experiments at any time. To this end, the preparation and configuration can be repeated and updated. To maintain an overview of the project, the information on every single experiment is kept together in a central table (file samples.tsv). One can further annotate the table entries with arbitrary additional information, such as the identity of the experimentalist (see Supplementary data).

#### Step 2: deriving reactivities from SHAPE-CE by QuShape

The CE raw data files are analyzed with the software QuShape [12]. This state-of-the-art tool largely facilitates the derivation of reactivity profiles based on the fluorescence profiles coming from CE sequencers. Importantly, files previously analyzed by QuShape may be added to benefit from the downstream pipeline.

#### Step 3: normalization and removal of outliers

Outliers, mostly representing RT intrinsic stops, are removed, set to −10, and the reactivities are normalized according to [34] (see the ‘‘Materials and methods’’ section). In brief, two normalization methods can be applied; for RNA under 300 nt, the highest 2% are considered as outliers, and reactivity values are normalized to the average of the 8% highest values. For longer RNA, values superior to the 75th percentile by over 1.5 the interquartile are removed, and the 10% remaining are averaged and used to normalize the reactivity. Importantly, reactivity files from any workflow, and most notably from high-throughput processes, such as SHAPE-MaP or DMS-MaPseq, can be fed into the pipeline at this stage. However, for consistency, one should check whether all the reactivity files used in the same project follow the same outlier removal and normalization rules.

#### Step 4: aggregation of replicates

IPANEMAP Suite then automatically gathers reactivity data obtained for the same RNA in the same probing conditions, thus aggregating data from replicates. It also concatenates reactivity values from elongations performed with different primers on the same RNA in the same condition (see the ‘‘Materials and methods’’ section). The average reactivity value for each nucleotide and its standard deviation are automatically calculated. If only one experiment is performed, the workflow will run with single data. The overall consistency of the replicates may be checked by calculating pairwise Pearson and Spearman correlation coefficients. The results are stored in the file but do not have any automated consequences. Nonconsistent replicates may be annotated as ‘‘discarded’’ in the samples.tsv file to ignore them in subsequent runs.

#### Step 5: modeling and analysis

##### RNA structure modeling by IPANEMAP

Models of the RNA secondary structure are generated using our software IPANEMAP. This tool uniquely allows the use of data from multiple probing experiments under different conditions and/or using different probes, as well as evolutionary information from MSAs. As described in the ‘‘Material and methods’’ section as well as in much more detail in the original publication [23], IPANEMAP aims to predict structures in best agreement with all reactivity and alignment input information as well as the thermodynamic RNA nearest neighbor model [39].

Using the pipeline’s graphical interface, the user can configure—and experiment with—arbitrarily many IPANEMAP prediction runs based on different combinations of datasets. As direct output, IPANEMAP predicts (see the ‘‘Materials and methods’’ section) best and second-best RNA secondary structures in dot–bracket format, which are furthermore visualized as reactivity-annotated structure drawings using VARNA.

##### Footprinting analysis

IPANEMAP Suite compares reactivities of different conditions and produces intuitively interpretable visualizations (see the ‘‘Materials and methods’’ section). Again, the pipeline allows configuring arbitrarily many comparisons and concrete visualization through its graphical configuration interface. Significant differences are reported in publication-quality files in the form of histograms and secondary structure representations. See also Fig. 4 from our examples.

**Figure 2.**
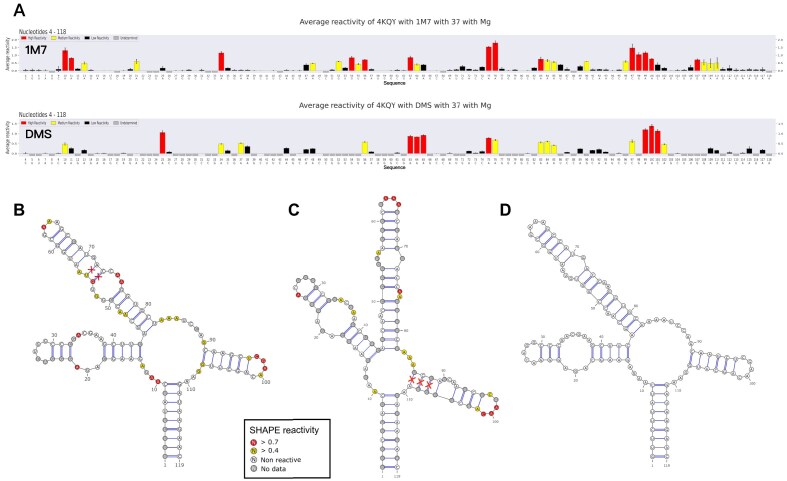
Structure modeling based on multiple probing datasets. (**A**) 1M7 (top) and DMS (bottom) reactivity profiles of *Bacillus subtilis yitJ* riboswitch. Secondary structure model colored with reactivities, obtained with 1M7 (**B**) and DMS (**C**) reactivity constraints. The red crosses mark base pairs that are predicted using 1M7 or DMS data but are not observed in the crystal structure. (**D**) Secondary structure model based on the integration of two probe datasets, which exactly matches the crystal structure. The respective prediction performances of panels (B), (C), and (D) were calculated as 0.93, 0.92, and 1.00 in terms of the widely used F1 score [40], the harmonic mean of recall and precision of base pairs with respect to the crystal structure. (See Supplementary data for the configuration of the structure modeling based on multiple profiles.)

**Figure 3.**
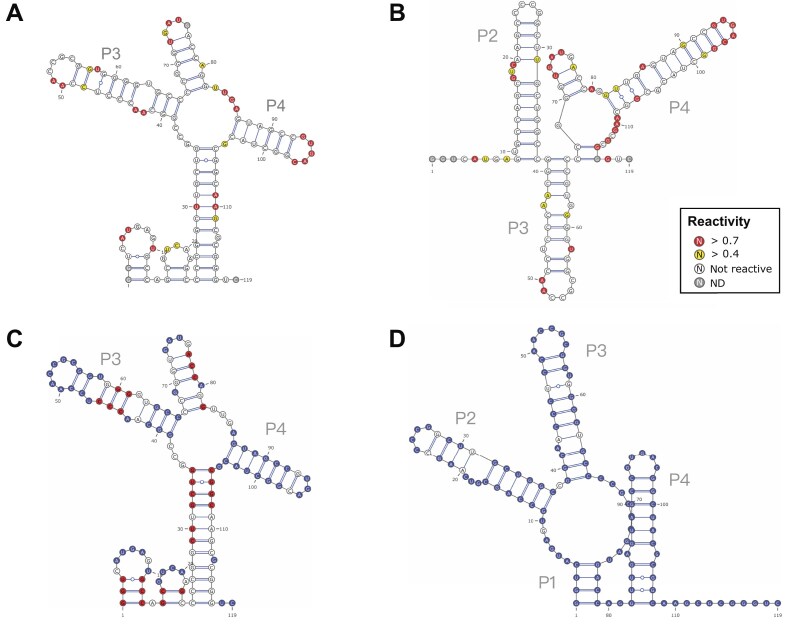
Structure model refinement by using MSA. (**A**) *Mycobacterium* SAM-IV secondary structure model derived using 1M7 reactivity constraints. (**B**) The secondary structure model was predicted using DMS reactivity constraints. (**C**) Refined model generated using the combination of 1M7 and DMS reactivity constraints; the color annotation represents the result of the comparison with the crystal structure (PDB ID: 6WLR). The nucleotides in blue are predicted as in the crystal structure, those in red are different but are in double strands in both structures, while nucleotides in white have different statuses in the two structures. (**D**) Further refined model integrating the information from 1M7 and DMS probing with phylogenetic data; the color annotation represents the result of the comparison with the crystal structure (PDB ID: 6WLR). The respective accuracies (F1 scores; see Fig. 2) of the predictions in panels (A), (B), (C), and (D) were calculated as 0.405, 0.719, 0.319, and 0.923, respectively, the latter quantifying a high similarity to the crystal structure (PDB ID: 6WLR).

**Figure 4.**
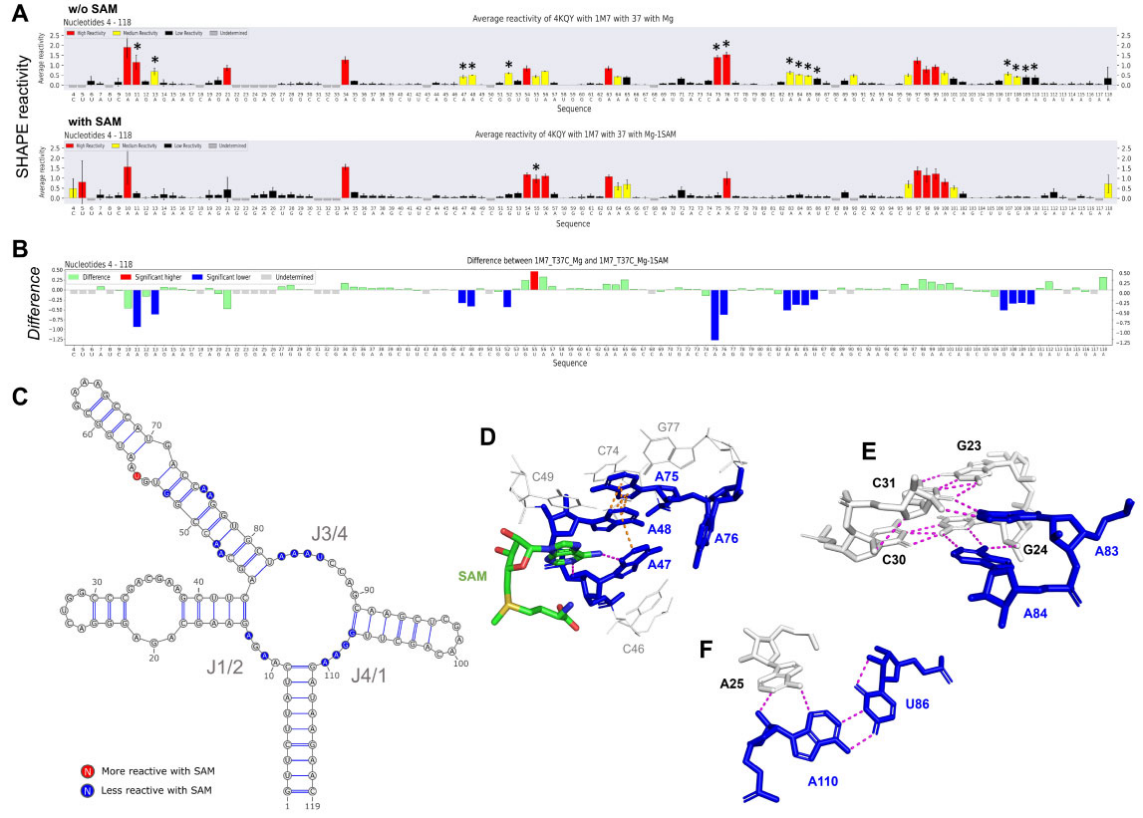
SHAPE analysis of the structural switch of *B. subtilis yitJ* SAM-I aptamer in the presence of *S*-adenosyl-methionine (SAM). (**A**) Normalized SHAPE reactivity profiles in the absence (top) and the presence (bottom) of the ligand (SAM). Asterisks indicate nucleotides with significant differential reactivity. (**B**) Histogram of the differential SHAPE reactivities (Δ*R*) calculated by subtracting the SAM-bound state profile from that of the unbound state. Positions exhibiting significant differential reactivities (see the ‘‘Materials and methods’’ section) are colored in blue for nucleotides less reactive in the presence of the ligand and in red for those more reactive in the presence of the ligand. (**C**) Secondary structure model colored with significant differences. Single-stranded junctions (J1/2, J3/4, and J4/1) are known to be less reactive upon SAM binding [41]. (**D**) SAM interaction with A47 induces pi-stacking (orange dotted lines) between nucleotides A47–A48–A76, which enhance the stability of those nucleotides. Base triple interactions formed after SAM binding (**E**) A83 and A84 with base pairs G23:C31 and G24:C30, respectively, and (**F**) A25 with base pair U86:A110 [42]. Hydrogen bonds are represented by magenta dotted lines. (See Supplementary data and Supplementary Fig. S5B for configuration of the footprint analysis.)

A typical use case of footprinting conditions would be to determine a protein or a small molecule binding site on an RNA, or to evaluate the RNA structural rearrangement upon changing experimental conditions, such as, temperature, ionic conditions, and pH. It can also be used to understand the impact of modifications, such as punctual mutations or trimming of the sequence.

##### Secondary structure model comparison

Finally, we provide functionality to visually compare two structure models, which is especially instrumental in the case of medium-sized or long RNAs. Given two secondary structures in dot–bracket notation (dbn files), our comparison tool generates two output files visualizing the structural differences by color annotations. The first highlights the nucleotides with a common structure, while the second distinguishes three categories that are color-encoded: the nucleotides involved in the same structures (blue by default), those involved in different structures but paired in both models (red by default), and those in a different pairing status in both models (white by default).

### IPANEMAP Suite use cases and example results

IPANEMAP Suite provides valuable tools for RNA structure investigation and generates numerous plots and figures to streamline data analysis and interpretation, minimizing the need for time-consuming manual plotting.

To showcase the fundamental, unique key features of the pipeline, namely the modeling based on multiple probing data, two ‘‘benchmark’’ RNAs with crystal structure models were probed under various conditions. Namely, we probed the SAM-IV aptamer from *Mycobacterium* and the *yitJ* SAM-I riboswitch from *B. subtilis* with previously solved X-ray crystallographic structures (PDB IDs: 6WLR [43] and 4KQY [42], respectively). Please note that datasets for those examples are available at https://github.com/Sargueil-CiTCoM/ipasuite.

#### Integration of multiple sets of data improve secondary structure prediction

The *yitJ* SAM-I aptamer was probed with 1M7 (Fig. 2A, top) and DMS (Fig. 2, bottom) in the absence of the ligand. Three RNA structure models were generated based on

- 1M7 (Fig. 2B),
- DMS probing data (Fig. 2C) separately, and
- using the multi-probing integration of IPANEMAP (Fig. 2D).

Here, IPANEMAP Suite’s project-based design allows us to easily produce the different models from the same project by configuring different IPANEMAP modeling setups in the graphical interface.

Figure 2 illustrates that neither of the models obtained using only one probing profile is correct. Due to the unique multi-probing features of IPANEMAP Suite, we show here that the integration of probing data using two different probes fundamentally improves the prediction of the *yitJ* SAM-I riboswitch, as the model obtained with both datasets exactly agrees with the secondary structure derived from the crystal structure.

#### Further refining structural models by integrating phylogenetic data

The *Mycobactetium* SAM-IV aptamer was subjected to DMS and 1M7 SHAPE probing. IPANEMAP was run using the 1M7 reactivity profile alone (Fig. 3A), the DMS reactivity profile alone (Fig. 3B), 1M7 and DMS datasets conjointly (Fig. 3C), or 1M7 and DMS datasets in conjunction with phylogenetic data (Fig. 3D). Different runs of IPANEMAP are easily configured in the graphic interface of IPANEMAP Suite, and the models obtained may be readily compared in regard to the secondary structure extracted from the PDB 3D structure (Fig. 3C and D). The crystal structure (6WLR) features four stems termed P1, P2, P3, and P4. The model obtained with 1M7 only predicts P3, P4, and two short spurious hairpins (Fig. 3A); the DMS-informed model features P2, P3, P4, and one short false stem (Fig. 3B). While the model obtained using both 1M7 and DMS reactivities (Fig. 3C) is similar to the one obtained with 1M7 only (Fig. 3A), the model using both datasets and the covariation information is very close to the secondary structure derived from the crystal structure (Fig. 3D). The same modeling strategy was applied to various benchmark RNAs of various lengths and functions, and belonging to various organisms: *Thermatoga maritima* ribonuclease P (347 nucleotides, PDB ID: 3Q1Q), *Sacharomyces cerevisiae* U1 snRNA (568 nucleotides, PDB ID: 7OQC), *Syntrophothermus lipocalidus* PRPP riboswitch (107 nucleotides, PDB ID: 6DNR), and *Escherichia coli* tRNAPhe (76 nucleotides, PDB ID: 3L0U). The accuracy (F1 score) for each prediction is reported in Table 1, and the structure models obtained with 1M7 and DMS datasets conjointly with phylogenetic data are presented in Supplementary Fig. S1 compared to the secondary structure derived from the crystal structure. Although the relative accuracy of the modeling using the various constraints may vary, the prediction, including both datasets and the phylogenetic information, is always the most similar to the secondary structure derived from the crystal structure. Furthermore, Supplementary Fig. S1 shows a remarkable accuracy of the models obtained for benchmark RNAs, including long and complex ones, such as RNase P or U1 snRNA. This demonstrates that IPANEMAP’s unique ability to integrate several datasets and phylogenetic information can be decisive in obtaining accurate models.

**Table 1.** Modeling accuracy of benchmark RNAs using 1M7 and DMS datasets, and phylogenetic data as F1 scores [40]

PDB	DMS	1M7	1M7 and DMS	1M7 and DMS and Phylogeny
3Q1Q	0.667	0.527	0.677	0.735
7OQC	0.450	0.468	0.466	0.731
6DNR	0.815	0.786	0.815	0.863
3L0U	0.955	0.977	0.955	0.977

*Thermatoga maritima* ribonuclease P (347 nucleotides, PDB ID: 3Q1Q), *Sacharomyces cerevisiae* U1 snRNA (568 nucleotides, PDB ID: 7OQC), *Syntrophothermus lipocalidus* PRPP riboswitch (107 nucleotides, PDB ID: 6DNR), and *E. coli* tRNAPhe (76 nucleotides, PDB ID: 3L0U) were probed with 1M7, DMS, and MSA obtained from Rfam [45]. RNA structures were modeled using 1M7, DMS, 1M7, and DMS datasets or 1M7 and DMS in conjunction with phylogenetic data using IPANEMAP Suite.

#### Footprinting analysis of SAM-I aptamer structural switch upon binding its ligand

To illustrate the usefulness of the footprint analysis tool using the IPANEMAP Suite pipeline, we performed SHAPE probing on the *B. subtilis yitJ* SAM-I riboswitch in the presence and absence of its ligand, the SAM (Fig. 4A). SHAPE probing has been widely used for aptamer characterization, including for the identification of nucleotides involved in ligand recognition and in tertiary structure formation [41, 44]. To compare the two probing profiles, IPANEMAP Suite automatically generates a SHAPE differential histogram, highlighting nucleotides with significant reactivity differences (Fig. 4B). These differences are also automatically displayed on the RNA secondary structure model (Fig. 4C).

As previously described for the SAM-I aptamer [42], the IPANEMAP Suite footprint analysis readily highlights significant reactivity decreases upon SAM recognition, allowing for the rapid identification of nucleotides involved in direct and indirect ligand contacts (Fig. 4D), as well as in the ligand-induced structural switch (Fig. 4B, D, E, and F). By automating the plotting process, the IPANEMAP Suite footprint tool eliminates the need for time-consuming manual efforts. More importantly, IPANEMAP Suite ensures the consistency and the data treatment objectivity essential to enable reliable conclusions to be drawn from such experiments.

## Conclusion

IPANEMAP Suite is an integrated workflow that organizes the user journey from the raw data to the representation of the secondary structure, while facilitating even advanced modeling strategies. It does not necessitate any specific expertise either in RNA structure or in computer science or bioinformatics. All along the pipeline, files representing intermediate results are stored in identified folders. Initialization of the project requires unambiguously naming the raw data files, and this denomination is kept throughout the process. IPANEMAP Suite allows you to seamlessly work on long RNA by automatically concatenating results obtained with different elongations covering the same RNA. These features avoid many file manipulations and thus prevent potential errors. It features a new version of IPANEMAP that can be informed by various structure probing datasets but also integrates the covariation information contained in an MSA. As shown above, the inclusion of multiple datasets and/or alignments may be critical to obtaining an accurate model. The footprint option facilitates comparing the reactivities obtained on the same sequence in different contexts. This enables, for instance, easily pinpointing a protein binding site on your favorite RNA. Although such analysis is routinely performed in labs studying RNA structure, this has not been implemented in any automated workflow to date. In addition to ease of use, IPANEMAP Suite provides the indispensable systematic routine to the footprinting approach. Indeed, drawing conclusions from such approaches requires a strict consistency in both the experimental and the analytical workflow. IPANEMAP Suite allows for a systematic and objective treatment of the raw data file, while offering flexibility through parameters that can be project-wide configured by the experimentalist. Finally, the comparison tool allows for an effortless visual comparison of two structure models even for medium-sized to long RNAs.

### Comparison with other workflows

Two other analytical workflows propose a continuous pipeline from the raw reactivity files obtained from RNA structure probing experiments to a secondary structure model. RNAthor features a slightly more sophisticated algorithm for the removal of the outlier values and offers a statistical analysis to evaluate results consistency throughout replicates [25]. RNAprobe [24] allows for modeling with various RNA prediction software, and notably with ShapeKnots, including the interesting possibility to predict pseudoknots [18]. In addition to the secondary structure model colored with reactivity, RNAprobe may also color a 3D structure model provided by the user according to nucleotide reactivities. While IPANEMAP Suite supports a project-based workflow, which enforces the traceability from raw data to the model, the competing tools are only available as web servers and do not integrate the entire workflow starting from the raw data. In addition, IPANEMAP Suite offers advanced functionality, such as the possibility to carry out secondary structure modeling driven by different datasets at once, or the footprinting analysis.

Over the last few years, many high-throughput experimental [29, 46–49] and computing pipelines [21, 50, 51] have been developed. They offer many possibilities to analyze high-throughput structure probing, and notably, Superfold can also take into account different sets of probing data for secondary structure probing, although it can only be implemented with three different SHAPE reagents [21]. IPANEMAP Suite can take into account data coming from both traditional and high-throughput structure probing, eliminate the risk of error when handling files, and allow organizing data and modeling in a consistent and project-oriented way. Finally, IPANEMAP Suite offers the unique possibility to model RNA secondary structure, integrating both multiple probing datasets and covariation data to further improve prediction accuracy. Future versions of IPANEMAP Suite will integrate principled metrics to assess the significance of covariations, allowing for more robust predictions [52].

## Supplementary Material

lqaf028_Supplemental_Files

## Data Availability

All software is made available for installation as Conda packages in channels Sargueil-CiTCoM or available in channel bioconda. The source code is accessible in Git repositories: IPANEMAP Suite: https://github.com/Sargueil-CiTCoM/ipasuite IPANEMAP: https://gitlab.inria.fr/amibio/ipanemap VARNA: https://github.com/yannponty/VARNA ViennaRNA: https://github.com/ViennaRNA/ViennaRNA The code and data have been deposited in Zenodo: https://zenodo.org/records/14936737. A figure showing refined models integrating the information from 1M7 and DMS probing conjointly with phylogenetic data, and a tutorial are available separately in Supplementary data.
