# Supplementary material for: IPANEMAP Suite: a pipeline for probing-informed RNA structure modeling
Source: NAR Genom Bioinform. 2025 Mar 25;7(1):lqaf028. doi: 10.1093/nargab/lqaf028 (PMC11934922; doi:10.1093/nargab/lqaf028)
Supplement: lqaf028_Supplemental_Files [file lqaf028_supplemental_files.zip › IPANEMAP_Suite_tutorial.pdf]

IPANEMAP Suite tutorial

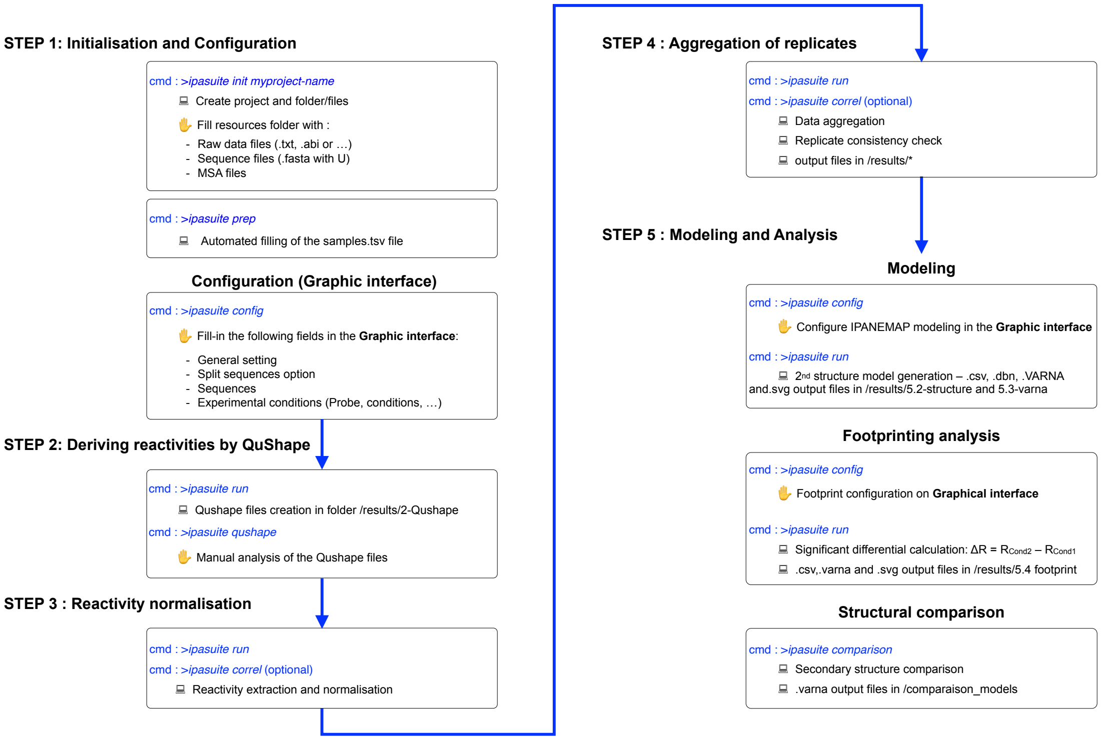

Figure 1: IPANEMAP Suite Workflow.

1 Initialisation of projects and configuration

The project is initialised by launching the command `ipasuite init myproject-name` (Figure 1). This creates a series of files and folders necessary to run the project (Figure 2). In the first step the capillary sequencer files are analysed by Qushape [1], to this end, they are placed in the `/myproject-name/ressources/raw_data` folder created at the project initialisation. Files should be named as described in Section Input files to allow their automated integration into the project. Otherwise the `sample.tsv` file found in `myproject-name` folder needs to be filled manually. The sequences fasta files are placed in `myproject-name/resources` folder, note that several different sequences can be used in the same project. The path to each sequence fasta file must be declared in the graphic interface. Then launch the command `ipasuite prep` (Figure 3). The relative file paths for the CE raw data files, reference files, or pre-treated QuShape output files are automatically populated when filenames follow the specified format. The resources folder can be populated with new files all along the project progress, notably with raw data files from replicates or additional probing conditions. Then the `ipasuite prep` command needs to be re-run and a new samples.tsv file is generated each time.

If using the option to concatenate elongations from different primers on the same RNA ("subsequences"), the nucleotides number for the start and the end of reverse transcription should be filled in samples.tsv.

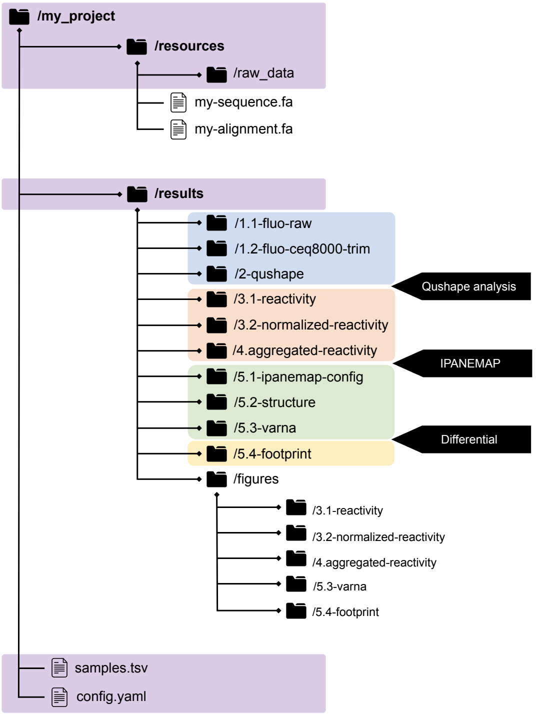

Figure 2: IPANEMAP Suite pipeline directory tree : Folder groups are color-coded based on their creation stage. Folders created after project initialization are highlighted in purple. Blue highlights folders created after raw data processing and QuShape files creation. Orange corresponds to folders containing reactivity files generated after Qushape analysis. Green corresponds to output folders created after ipanemap modelisation and yellow corresponds to figures folder, figures are created all along the pipeline for each step.

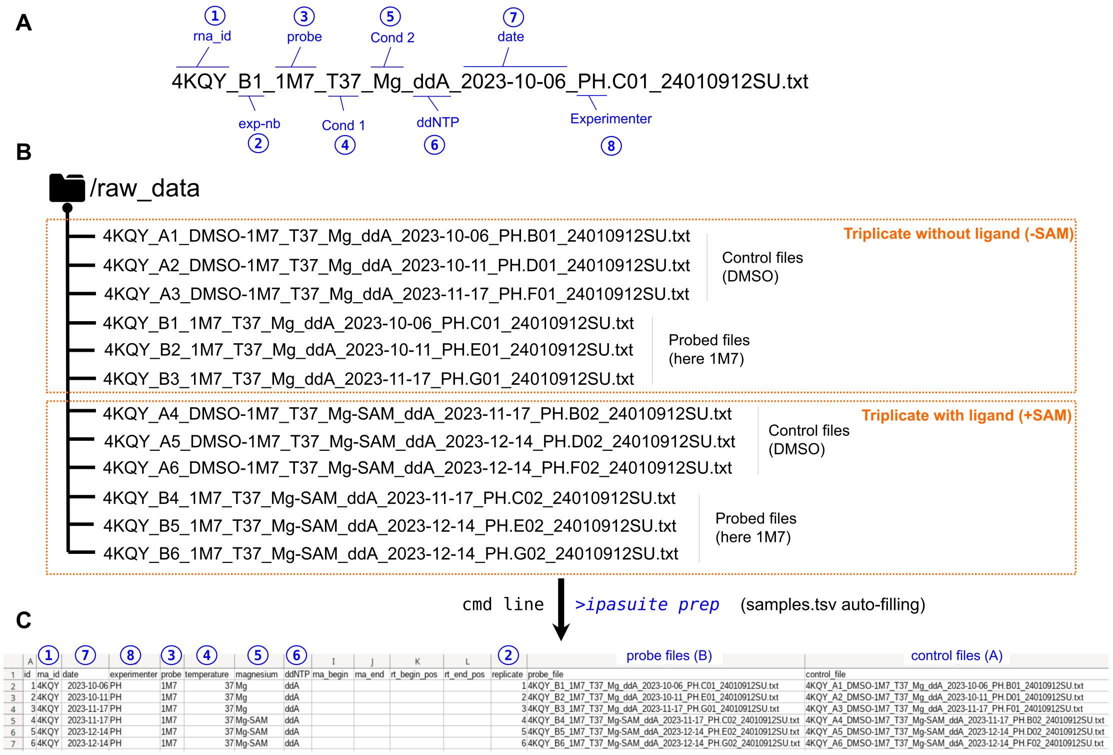

Figure 3: Streamlined data entry with autofill of the samples.tsv file : (A) Raw data files naming convention for automatic filling of the samples.tsv file. (B) Example of SHAPE-CE .txt input files provening from a CEQ8000 sequencer (C) Autofilled samples.tsv generated by the command `ipasuite prep`.

Input files

RNA sequences and RNA multiple sequence alignment files should be in fasta format (.fas). Although input files can be named arbitrarily, we have set a naming convention allowing for direct and automated integration into the pipeline (Figure 2) :

SHAPE-CE files should be placed in the folder `myproject-name/raw data` and named as follows:

- probed file : `RNAid_Bexp-nb_probe_cond1_cond2_ddNTP_date_experimenter.additional-information.txt`
- control file : `RNAid_Aexp-nb_DMSO-probe_cond1_cond2_ddNTP_date_experimenter.additional-information.txt`

RNAid is the name of the RNA of interest, A and B correspond to control (DMSO) and probed (SHAPE) reactions respectively, they are immediately followed by the experiment number (exp-nb) which should be the same for the cognate A and B. We use the replicate number as the experiment number, cond1 and cond2 are two parameters of the conditions in which the experiment has been carried out. For instance the magnesium concentration and the temperature, ddNTP corresponds to the chain terminator used in the sequencing reaction. Conditions are optional entries and may be replaced by a "x" or any meaningless character in the file title. The fields "experimenter" and additional information are optional. Already treated Qhshape files may also be integrated in the pipeline by placing them in files should be placed in `myproject-name/ressources/raw data` folder and named as follows:

`RNAid_reagent_cond1_cond2_exp-nb.additional-information.qushapey`

Reactivity files from any experimental/analytical pipeline such as SHAPE-CE, CAFA, HiTrace, FAST, RiboCat, or HT SHAPEMaP, SHAPESeq or DMSMApseq files may be integrated in the workflow. Such files should be tab delimited .txt including the nucleotide number in the first column and its reactivity in the second. they should be placed in `myproject-name/ressources/raw data` folder and named as follows:

`RNAid_reagent_cond1_cond2_exp-nb.additional-information.map`

Warning : 1) Files should carry the extension .map even not withstanding the software that generates them 2) For consistency one should check if all the reactivity files used in the same project follow the same outliers removal and normalisation rules.

Input files may be added in the course of a project, update of the samples.tsv file through `ipasuite prep` and if necessary (when adding conditions, IPANEMAP or footprint runs) through the graphic interface should be performed for the reactivity files to be integrated into the pipeline.

In IPANEMAP Suite, cond1 and cond2 are predefined as temperature and magnesium concentration respectively. These two parameters are frequently modified to get information about RNA tertiary structure [2], but the "conds" field may contain any useful information. File naming convention is not mandatory, but in case another nomenclature is used for naming the files, information necessary to process the data should be manually declared in the samples.tsv file. Similarly, a infinite number of conditions may be used, but they need to be manually entered in the samples.tsv file. Example files are available at https://github.com/Sargueil-CiTCoM/ipasuite

Configuration

Once initialised, the project is parametrized in the graphical interface launched by `ipasuite config`. The conditions and probes are entered in this interface, there is no limit to the number of sequences, conditions or probes used.

In the two last boxes IPANEMAP and footprint runs are configured, here again there is no limit to the number of runs that can be set-up and run indefinitely (Figure 5). Each IPANEMAP run is identified by a unique ID (pool_ID) ensuring traceability (Figure 4). Upon addition of new data files in an ongoing project, IPANEMAP Suite can be rerun, updating information in the graphic interface is necessary only if new sequences, probes or conditions are added, then `ipasuite prep` needs to be re-run.

Concatenation of results obtained by elongations from different primers on the same RNA

IPANEMAP suite offers the possibility to automatically concatenate results obtained from the same sequence using different primers. This allows to easily work with long RNA. To use this option, tick the `use subsequence` in the graphical interface, and enter the primers positions in the `sample.tsv` file.

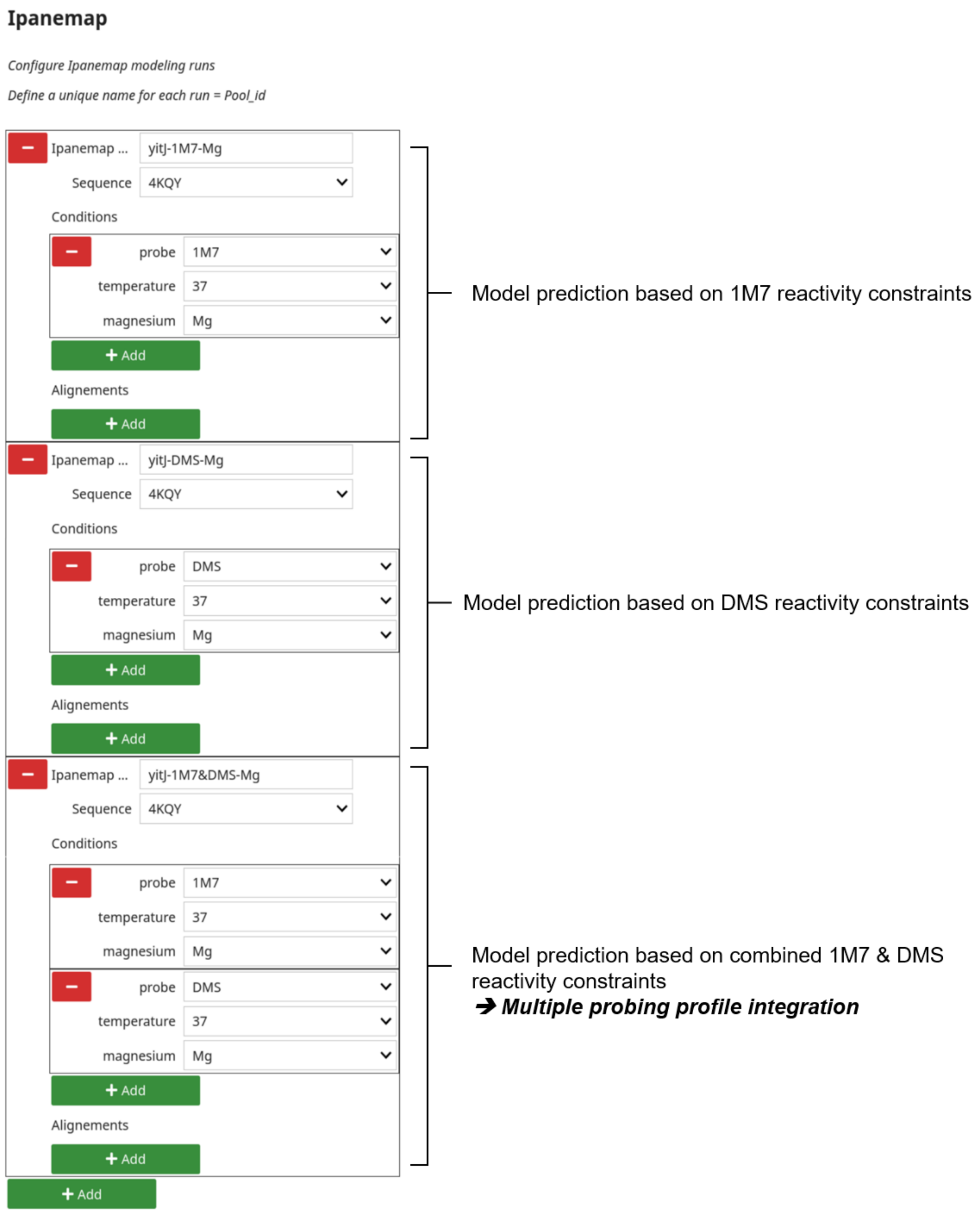

Figure 4: Configuration of the Multiple probing profiles integration option. The third IPANEMAP box at the bottom represents the model prediction configuration that incorporates both 1M7 and DMS reactivity constraints to guide the prediction process.

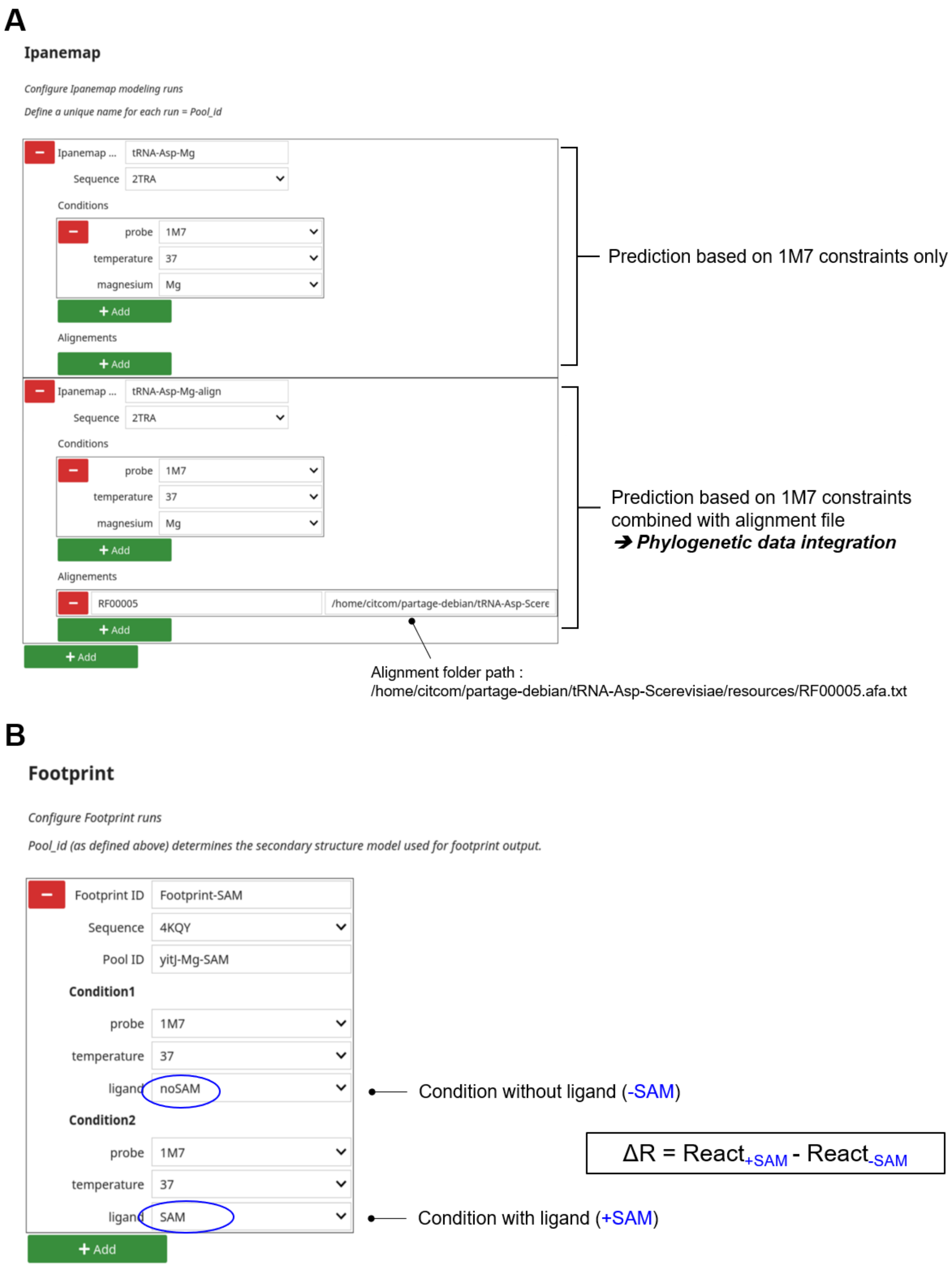

Figure 5: (A) Configuration of the phylogenetic data integration for secondary structure model prediction. The title for the alignment is arbitrary. In this example, the Rfam tRNA identifier RF00005 has been used. Key feature : Enter the full path to the MSA file. (B) Footprint analysis configuration. In this example, footprint is configured to highlight significant reactivity differences induced by ligand addition.

2 Deriving reactivities by QuShape

QuShape analysis can then be launched by `ipasuite qushape`. The capillary electrophoresis raw data files are analysed with QuShape according to the procedure described in [1]. The QuShape output files (.qushapey) are stored in the folder `myproject-name/results/2-qushape`. Importantly, files previously analysed by QuShape may be added in this folder to be implemented in the downstream pipeline. In case those files use a probe, or a condition that was not registered in the original configuration, `ipasuite config` needs to be re-run.

3 Normalisation and removal of outliers

The command `ipasuite run` then launches the pipeline until the end of the analysis as parametrized in the configuration interface. This command will process the data, model the structure and generate all the figures as described below.

Raw reactivity files are stored in `myproject-name/results/3-1-reactivity`, normalized reactivity files are stored into `myproject-name/results/3.2-normalized-reactivity` and are under a tab delimited format with the first column being the nucleotide number, the second the nucleotide identity and the sixth corresponding to the normalised reactivity value.

4 Aggregation of replicates and consistency check between replicates

Then mean reactivity observed of each replicates and the standard deviation are calculated the corresponding files are stored in `myproject-name/results/4-aggregated reactivity` , histogramms (.svg files) are generated and stored in `myproject-name/figures/4-aggregated-reactivity`. The default values for aggragating relicates are min_std=0.15, reactivity_medium=0.4 and reactivity_high=0.7. These parameters can be modified in the `myproject-name/config.yaml` file.

Before running the whole pipeline, it is possible to check data consistency. In addition to the reactivity files (see below), `ipasuite correl` analyse the replicates consistency by calculating pairwise Pearson and Spearman correlation coefficients for each conditions. The results are stored in `RNAid_reagent_cond1_cond2_consistency.csv` file, but do not have any automated consequences. The user can annotate non-consistent replicates as ‘discarded’ in the samples.tsv file (enter "yes" in the "discard" column) to ignore them in a subsequent run. Then, first type the command `ipasuite clean` to erase all processed data downstream /2-Qushape analysis, then run `ipasuite correl` or `ipasuite run` to re-process the data.

5 Modeling and Analysis

RNA structure modeling by IPANEMAP

IPANEMAP Suite then yields two secondary structure models for each run configured in the graphic interface. In the first box enter the Pool_id which unambiguously defines an IPANEMAP run (see above). Models are available under different file formats in `myproject_name/results/5.2-structure` and `myproject_name/results/5.3-varna` folder (Figure 1), as standard dot bracket file (.dbn) allowing to draw the secondary structure with most of the secondary structure visualisation software, as Varna session files (.varna) allowing the visualisation of the secondary structure coloured according to the reactivity, and finally as vector graphics svg files. Note that IPANEMAP runs may be configured at anytime and run launching the command `ipasuite run` which will not recalculate the reactivity but only execute the new IPANEMAP runs configured.

Footprinting analysis

For each footptint configured in the graphic interface (see above) IPANEMAP suite then yields results as `RNAid_footprint_foot-id.tsv` files which include the averaged reactivity of each nucleotide for each condition, their differences, the relative changes, p-values from a two-sided t-test, and significance results (.tsv files). The information is also presented in histograms (.svg files) and secondary structure figures (.svg and .varna files). All results are stored in the folder `myproject_name/results/figures/5.4-footprint`. The Pool_ID defines the structure model used for the secondary structure figures. Note that footprint runs may be configured at anytime

and run launching the command `ipasuite run` which will not recalculate the reactivity but only execute the new footprint runs configured.

Structural comparison

The pipeline also supports the comparison of secondary structures. The command `ipasuite comparison` can be executed in either the `myproject_name/` or `myproject_name/results/5.2-structure` folders to compare the secondary structures (dot bracket file (.dbn)) generated by IPANEMAP or located in any other folder containing such files. This process generates two Varna session files (.varna) in the `comparaison_models` folder based on the first selected structure, one file highlights the bases involved in the same structure in both models (in blue), while the other highlights both the bases involved in the same structure (in blue) and the paired bases that have different partner bases in the two structures (in red).

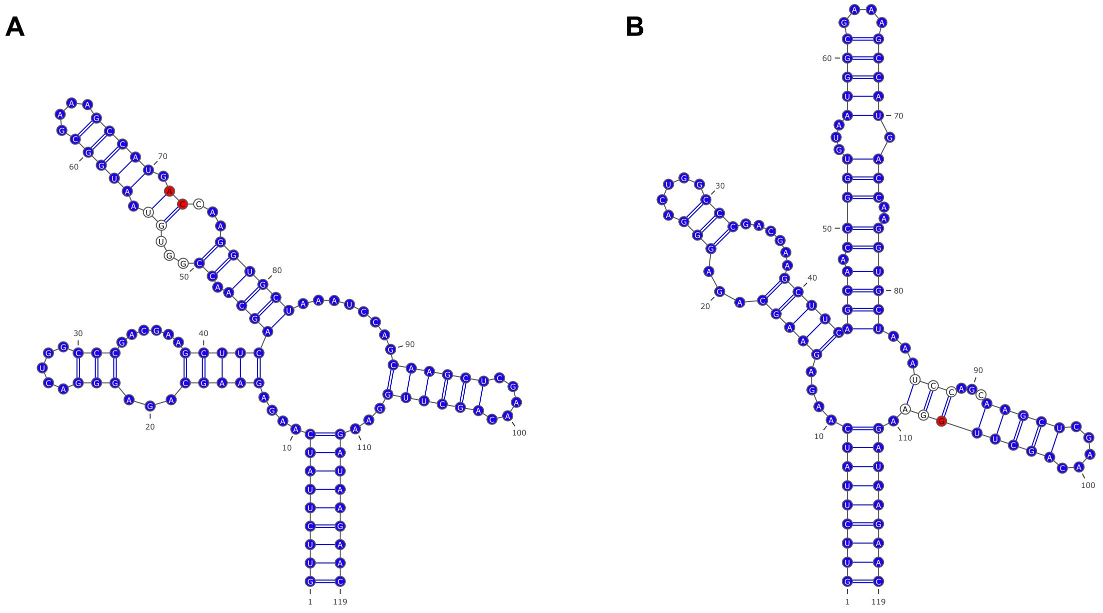

Figure 6: Examples of structural comparison : the nucleotides in blue are identical to those in the benchmark, the red ones differ but remain in a double strand in both structures, and the white ones have different statuses in the two structures.

References

[1] F. Karabiber, J. L. McGinnis, O. V. Favorov, and K. M. Weeks. QuShape: rapid, accurate, and best-practices quantification of nucleic acid probing information, resolved by capillary electrophoresis. *RNA*, 19(1):63–73, 2013.

[2] Grégoire De Bisschop, Delphine Allouche, Elisa Frezza, Benoît Masquida, Yann Ponty, Sebastian Will, and Bruno Sargueil. Progress toward SHAPE Constrained Computational Prediction of Tertiary Interactions in RNA Structure. *Non-Coding RNA*, 7(4), 2021.
