# Supplementary material for: IPANEMAP Suite: a pipeline for probing-informed RNA structure modeling
Source: NAR Genom Bioinform. 2025 Mar 25;7(1):lqaf028. doi: 10.1093/nargab/lqaf028 (PMC11934922; doi:10.1093/nargab/lqaf028)
Supplement: lqaf028_Supplemental_Files [file lqaf028_supplemental_files.zip › Supplementary figure.pdf]

Supplementary Information:
IPANEMAP Suite: A Pipeline for Probing-Informed RNA Structure Modeling

Pierre Hardouin[†], Nan Pan[†], Francois-Xavier Lyonnet du Moutier[†], Nathalie Chamond, Yann Ponty, Sebastian Will[*], Bruno Sargueil[*]

† These authors contribute equally. ∗ Corresponding authors

A – PDB : 3Q1Q

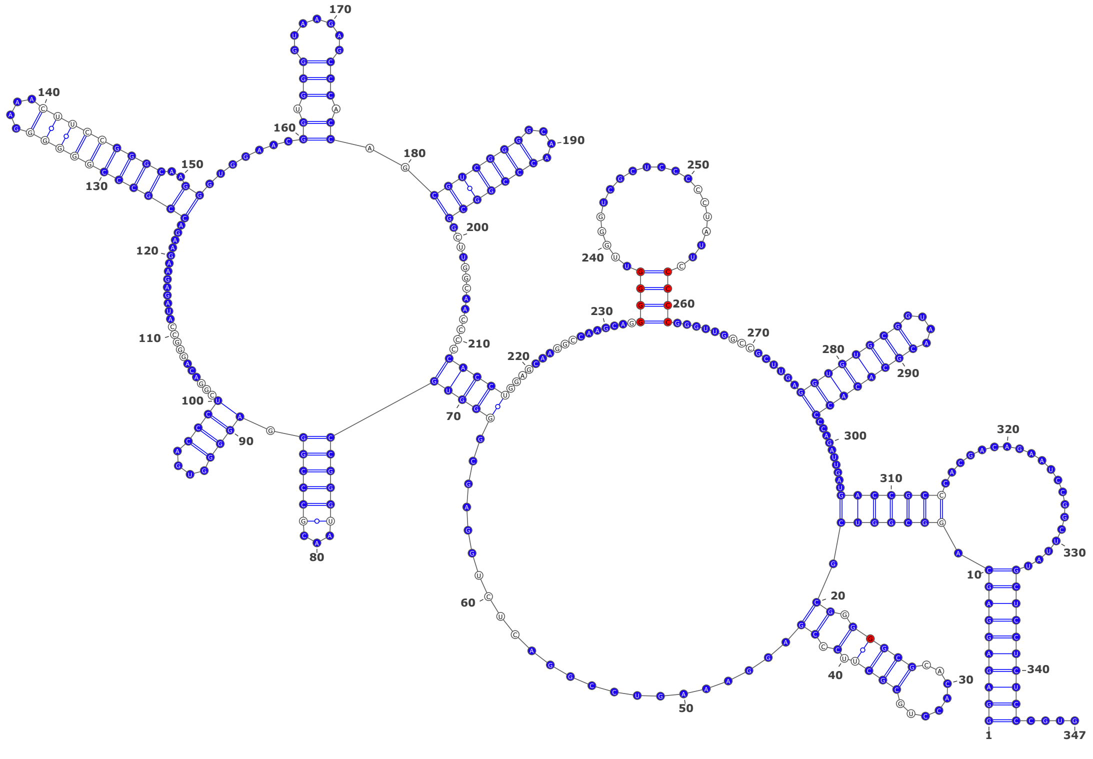

B – PDB : 7OQC

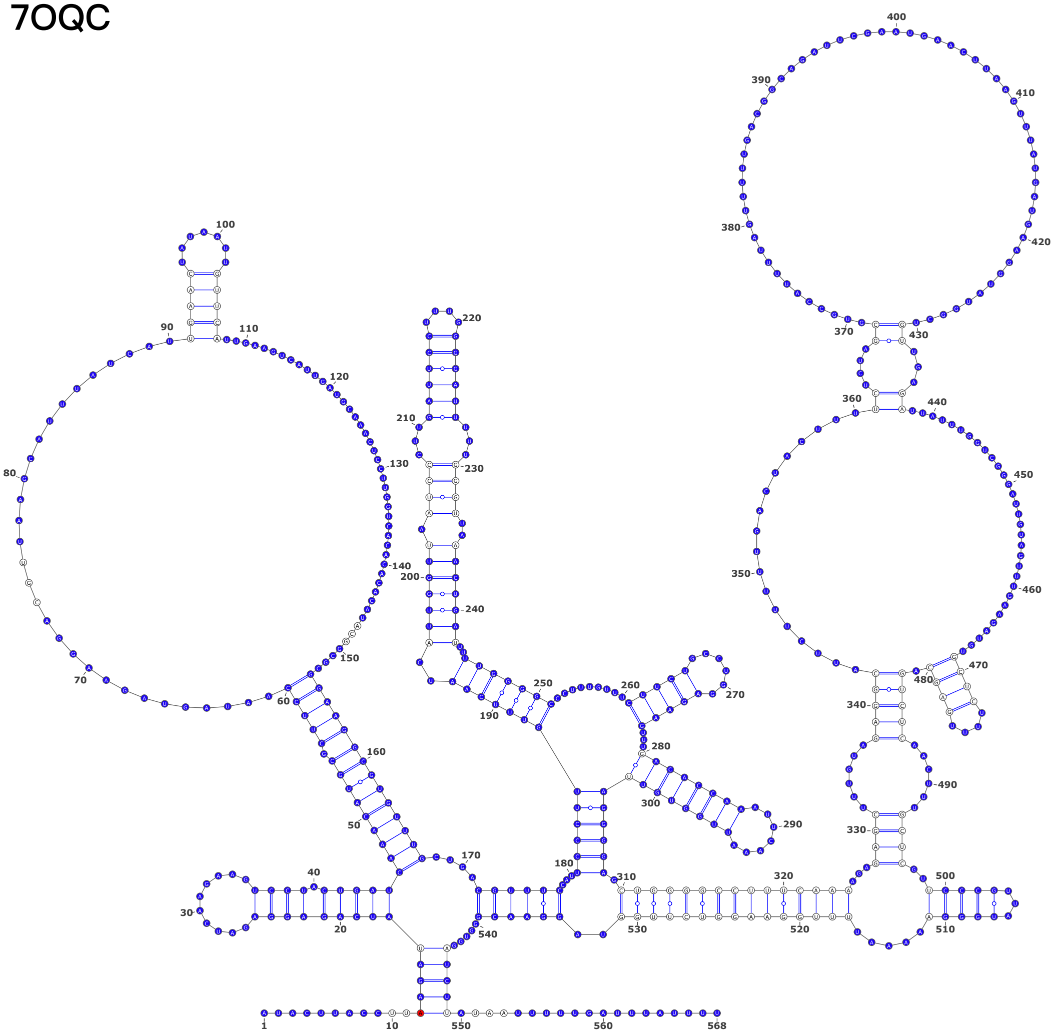

400
390
410
380
420
370
430
360
440
450
350
460
470
480
340
490
330
500
320
520
510
530
310
100
90
110
120
220
210
80
130
230
140
200
240
150
70
250
260
270
190
60
280
160
300
290
50
170
180
40
30
20
540
1
10
550
560
568

C – PDB : 6DNR

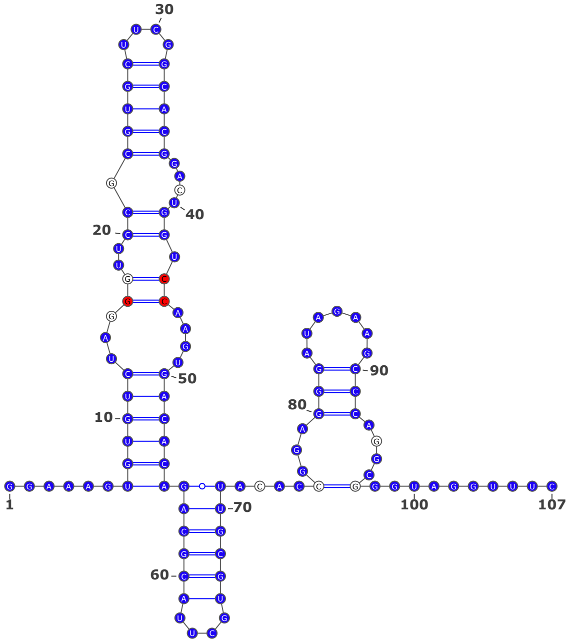

D – PDB : 3L0U

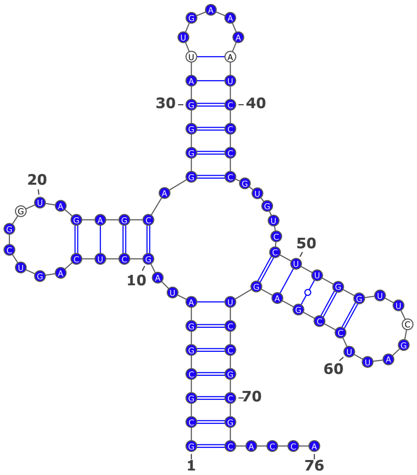

Figure S1: **Refined models integrating the information from 1M7 and DMS probing conjointly with phylogenetic data.**
The structures of (A) *Thermatoga maritima* ribonuclease P (347 nucleotides - PDB ID: 3Q1Q), (B) *Sacharomyces cerevisiae* U1 snRNA (568 nucleotides - PDB ID: 7OQC), (C) *Syntrophothermus lipocalidus* PRPP riboswitch (107 nucleotides - PDB ID: 6DNR), and (D) *Escherichia coli* tRNAPhe (76 nucleotides - PDB ID: 3L0U) were modeled with IPANEMAP Suite using 1M7 and DMS probing data, as well as a multiple sequence alignment (MSA) derived from the Rfam seed alignment. RNA models obtained were compared to the secondary structure model derived form the respective crystal structures. Nucleotides in blue are in the same structure in both models, those in red are in double strand in both models but not with the same partner and those in white do not have the same status in both models.
